# Characterization of the expression, promoter activity and molecular architecture of fibin

**DOI:** 10.1186/1471-2091-12-26

**Published:** 2011-05-26

**Authors:** Johannes Lakner, Christian Seyer, Thomas Hermsdorf, Torsten Schöneberg

**Affiliations:** 1 Molecular Biochemistry, Institute of Biochemistry, Medical Faculty, University of Leipzig, Johannisallee 30, Leipzig, 04103, Germany

## Abstract

**Background:**

Fibin was initially discovered as a secreted signal molecule essential for pectoral fin bud initiation in zebrafish. Currently, there is little information about the molecular architecture and biological relevance of fibin in humans and other mammals.

**Results:**

Fibin is expressed in cerebellum, skeletal muscle and many other embryonic and adult mouse tissues suggesting not only a role during embryonic development but also in adult functions. A 2.5-kbp genomic sequence fragment upstream of the coding sequence is sufficient to drive and regulate fibin expression through stimulation by glucocorticoids, activators of the protein kinase C signalling pathways and manganese ions. Fibin is an evolutionarily conserved protein, carries a cleavable signal peptide (amino acids 1-18) and is glycosylated at Asn^30^. The two conserved cysteines participate in intermolecular disulfide bond and multimer formation. Although fibin displays all features of a secretory protein, it is mostly retained in the endoplasmic reticulum when heterologously expressed.

**Conclusion:**

Fibin is functionally relevant during embryogenesis and adult life. Its expression is regulated by a number of cellular signalling pathways and the protein is routed via the secretory pathway. However, proper secretion presumably requires an unknown covalently-linked or associated co-factor.

## Background

Fibin, a secreted protein, was recently described as a novel growth factor found in various embryonic tissues of zebrafish and in the forelimb buds of mouse embryos [1]. The fibin sequence exhibits no homology to any known proteins. In zebrafish, the functional knockdown of fibin resulted in an absence of pectoral fins [1]. There are a number of factors involved in limb development; among them retinaldehyde dehydrogenase type 2 [2], the fibroblast growth factors fgf16 [3], fgf24 [4] and the transcription factor tbx5 [5]. Mutations in the tbx5 gene cause the Holt-Oram syndrome, which is characterized by defects of the upper limbs and heart [6]. Interestingly, fibin induces the expression of tbx5 via an unknown signalling cascade [1]. The findings suggest that fibin is essential for the pectoral fin bud formation during embryogenesis. However, human hereditary diseases caused by mutation of the fibin gene are not known yet.

Fibin attracted our attention because fibin mRNA levels were increased in the kidneys of newborn mice with X-linked nephrogenic diabetes insipidus [7]. Further, in-depth screening of public sources revealed that fibin appears to be highly expressed not only in embryonic stages, but also in a number of tissues in postnatal and adult stages, which implicates functions after embryonic ontogenesis. In the present study, we provide comprehensive data on its evolutionary origin, structural conservation, expression profiles, expression regulation, cellular targeting and protein biochemistry which includes heterologous protein expression and refolding. Although fibin is routed through the endoplasmic reticulum (ER) we found no significant evidence of secretion. We show that fibin is posttranslationally modified and forms dimers when heterologously expressed. Our data suggest that fibin may require yet unknown accessory proteins for proper function.

## Results

### Genomic organization, expression and expression regulation of fibin

**Figure 1:**
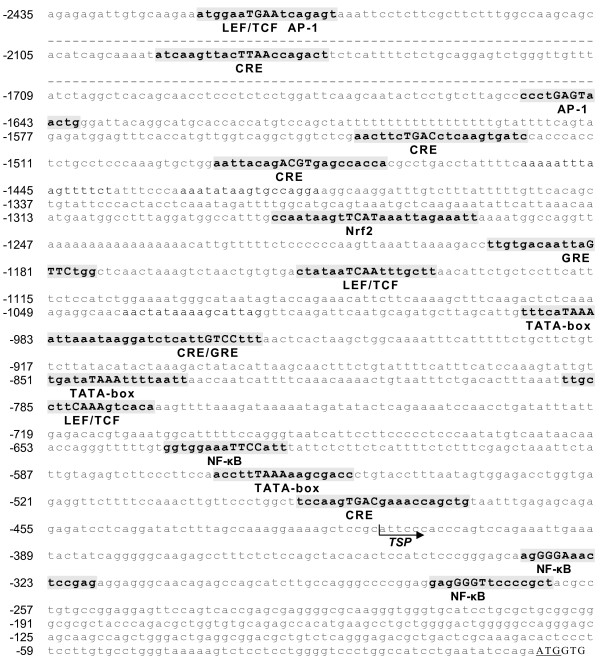
**Putative *cis*-acting elements of the 5'-flanking region of the human fibin gene**. Grey boxes indicate the transcription consensus-binding sequences of glucocorticoid-responsive and -related elements (GRE), cAMP responsive elements (CRE), LEF/TCF, NF-κB, AP-1, Nrf2 and TATA binding protein (TATA) factors. EST alignments (see Additional file 1, Figure S1) indicated more than one transcriptional start site (TSP). TSP indicates the most frequently 5'-end of ESTs. The translation start codon is underlined.

Initial investigations mining public sequence data sources (EST data bases) revealed that human and mouse fibin transcripts are present in a number of different tissues, such as bones, kidney and lung, and also during several ontogenetic stages (see http://www.ncbi.nlm.nih.gov/UniGene/ESTProfileViewer.cgi?uglist=Hs.712718; http://www.ncbi.nlm.nih.gov/UniGene/ESTProfileViewer.cgi?uglist=Mm.291809). Comparison of fibin mRNA and genomic sequences in humans and mice provided no indication of transcript splicing or editing. More detailed information of the genomic structure and transcript length are shown in Additional file 1, Figure S1. To map putative *cis*-acting elements in the 5' flanking region the MatInspector online software was used. We identified several binding sites for the glucocorticoid receptor, cAMP responsive element protein, NF-κB and Wnt-activated LEF/TCF transcriptional factors (Figure 1).

**Figure 2:**
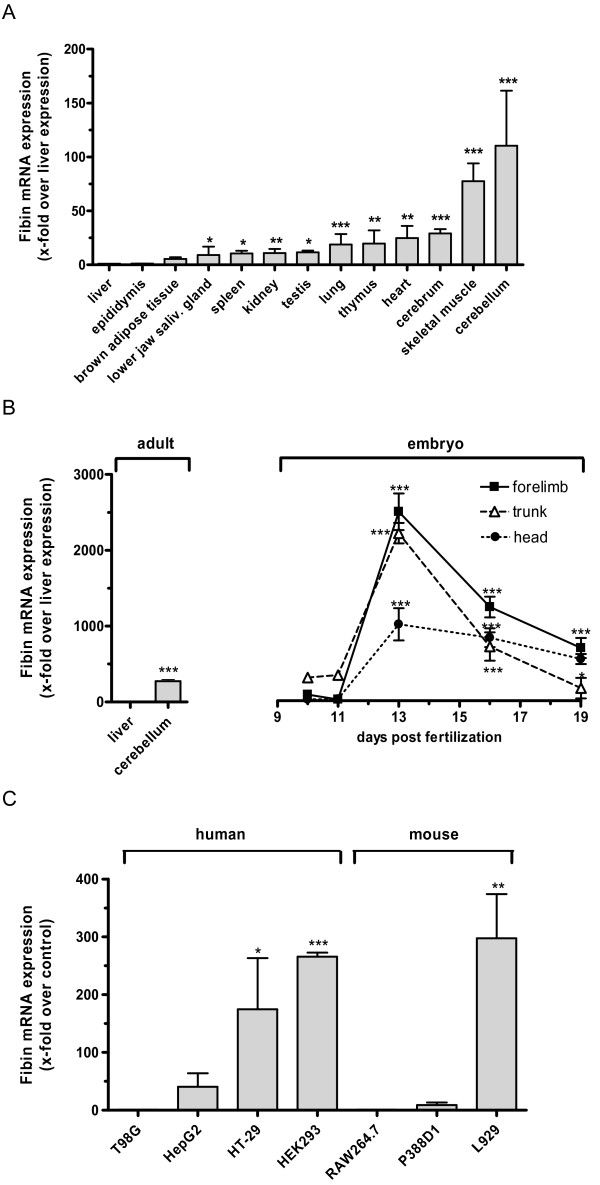
**Fibin mRNA expression estimated by qPCR**. Total RNA was extracted either from several mouse tissues or cell lines and used for reverse transcriptase reaction. qPCR was carried out with transcript-specific primers pairs. (A) Fibin mRNA expression of tissues from three adult mice is given as x-fold over fibin expression in liver. Data are shown as means ± SEM from three different tissue and cell preparations. (B) At indicated days after fertilization embryos were dissected from uterus. Trunks, forelimbs and heads of day 10 and 11 were individual pools prepared from 20 mouse embryos, and therefore not marked with error bars. Data of day 13 were generated by three pools for each body part consisting of 3-5 individuals. For day 16 and 19 the body parts of three individuals were prepared independently. Data of days 13, 16, 19 are shown as means ± SEM. To compare fibin expression between embryonic and adult tissues qPCR was performed together with samples prepared from adult liver and cerebellum. (C) Fibin expression in human and murine cell lines is given as x-fold over expression levels in T98G cells and RAW264.7 cells, respectively. Data are shown as means ± SEM from three independent cell preparations. Significant differences are marked with *p < 0.05, **p < 0.01, ***p < 0.001 using the Student's unpaired t-test.

To experimentally substantiate EST data, we performed qPCR studies with cDNA of various tissues and at different mouse embryonic stages in relation to β_2_-microglobulin. As shown in Figure 2A, increased fibin expression was found in the cerebellum and skeletal muscle. High expression levels of fibin mRNA were not only present in limbs of the mice at days 13 and 16 but also in the trunk (Figure 2B). Overall fibin expression was higher in fetal than adult tissues.

Next, we screened several human and mouse cell lines for possible fibin expression. Significant mRNA levels were found in HEK293, HT29, HepG2, and L929 cells (Figure 2C), whereas T98G and RAW264.7 cells presented very low fibin mRNA levels. Other human (LN405, K562, U937), mouse (P815, WEHI3B, X63) and rat (RBL2H3) cell lines showed no detectable fibin mRNA (not shown).

**Figure 3:**
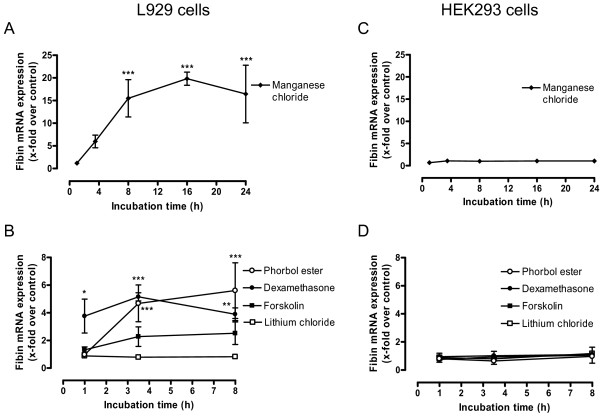
**Effects of additives on fibin expression in L929 and HEK293 cells**. L929 or HEK293 cells were incubated with (A) 300 μM MnCl_2 _or (B) with either 10 mM lithium chloride, 25 μM forskolin, 10 μM dexamethasone, or 10 μM phorbol ester (final concentrations) for indicated times. Then, samples were analyzed by real-time PCR. Data are given as means ± SEM of x-fold stimulation over controls of three independent experiments using the Student's unpaired t-test. Significant differences between experimental and control conditions are marked with *p < 0.05, **p < 0.01, ***p < 0.001.

To analyze fibin expression regulation, L929 and HEK293 cells were incubated with various compounds known to have effects on the expression of other genes. As shown in Figure 3A, manganese chloride significantly increased fibin expression in L929 cells. The highest levels were obtained after 16 hours. Dexamethasone and phorbol ester also significantly increased fibin expression but to a lesser extent than manganese chloride (Figure 3B). In HEK293 cells (Figures 3C and 3D), none of the compounds tested had any effect on the fibin mRNA level.

All attempts to quantify endogenous fibin expression in embryonic and adult tissues and cell lines at the protein level failed so far, probably because the protein amounts were below Western blot sensitivity (data not shown). Further, specific detection after enrichment of fibin from tissue (mouse and pig brains, 10 g and 200 g, respectively) and from cell line (HEK293, L929; 300-500 mill. cells) extracts by immunoprecipitation and immunoaffinity chromatography followed by Western blotting was unsuccessful. Both antibodies, against the whole protein and C terminus, have a detection limit of approximately 400 fmol fibin per lane (heterologously expressed in *E. coli *and purified) in Western blots indicating that amounts of fibin from tissue and cell extracts were below this concentration. Posttranslational proteolytic processing, as cause for the lack of immunodetection, can almost be excluded because posttranslational cleavage sites of prohormone convertases (PrpP 1.0 http://www.cbs.dtu.dk/services/ProP/ or conserved neighboured basic amino acid motifs were not identified.

**Figure 4:**
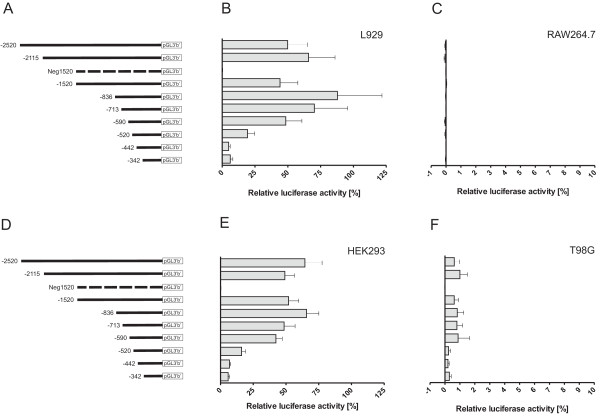
**Reporter assays with different fibin promoter lengths**. (A, D) Various lengths of human genomic sequences upstream of the fibin translation start were amplified and cloned into a pGL3 vector. Murine (B) L929 cells and (C) RAW264.7 cells as well as human (E) HEK293 cells and (F) T98G cells were transfected with the various promoter constructs. RAW264.7 cells and T98G cells served as the respective mouse and human negative controls because no endogenous fibin-mRNA expression was found (see Figure 2C). Luciferase activity was measured 48 h later and normalized to luciferase activity driven by an SV40 promoter with average values of 2,483 cps (L929 cells), 21,975 cps (HEK293), 12,896,311 cps (RAW264.7 cells), and 147,769 cps (T98G cells). Data are given as mean ± SEM of four to seven independent experiments.

To further address the mechanism of transcriptional regulation of fibin, human genomic sequences (up to 2.5 kbp upstream the translation start) were cloned into the firefly luciferase gene containing the pGL3 reporter vector (Figure 4A and 4D). The transcriptional start is located between -415 bp and -359 bp upstream of the translational start and there is no intron in the 5' UTR (for details, see Additional file 1, Figure S1). Maximum promoter activities in both, L929 and HEK293 cells were found with constructs containing -836 bp upstream of the genomic sequence (Figures 4B and 4E) indicating similar regulation of basal transcription in both cell lines. Mouse RAW264.7 cells and human T98G cells served as the respective negative controls because no endogenous fibin mRNA expression was found in either cell lines (Figures 4C and 4F).

**Figure 5:**
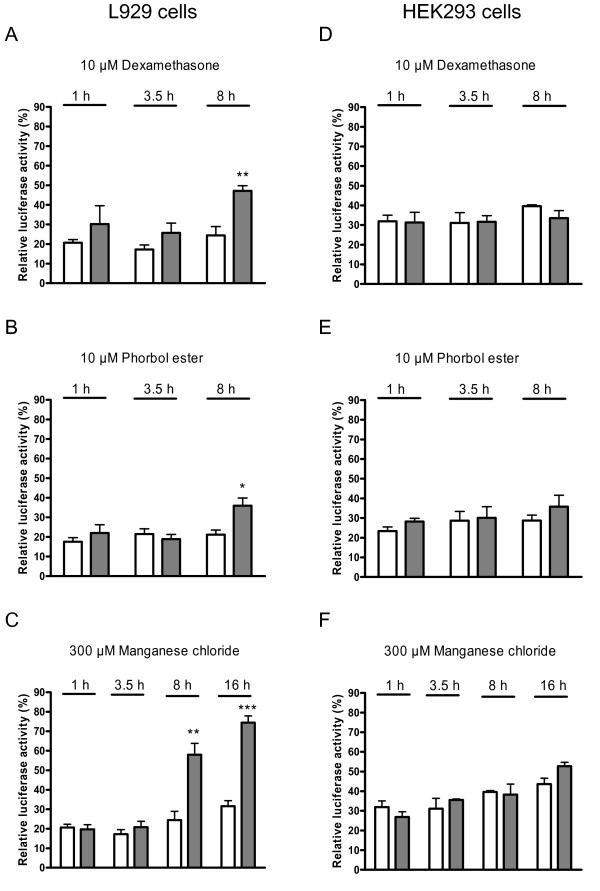
**Effects of additives on the activity of a transfected fibin-promoter construct**. L929 cells and HEK293 cells were transfected with the 2.5-kbp-fibin promoter construct. After 24 h, 10 μM dexamethasone, 10 μM phorbol ester, and 300 μM MnCl_2 _were added. Cells were lysed after the indicated time of incubation and luciferase activity was measured. Results (controls: white bars; compounds: grey bars) are shown as means of relative luciferase activity ± SEM of four (L929 cells) and three (HEK293 cells) experiments. Significant differences between sample and control conditions are marked with *p < 0.05, **p < 0.01, ***p < 0.001 using the Student's unpaired t-test.

To analyze whether the 2.5-kbp-promoter construct contains regulatory sites, transfected L929 and HEK293 cells were incubated with dexamethasone, phorbol ester and manganese chloride. As shown in Figure 5, significant regulation of luciferase activities by these three compounds was found in L929 cells but not in HEK293 cells.

### Subcellular distribution of fibin

**Figure 6:**
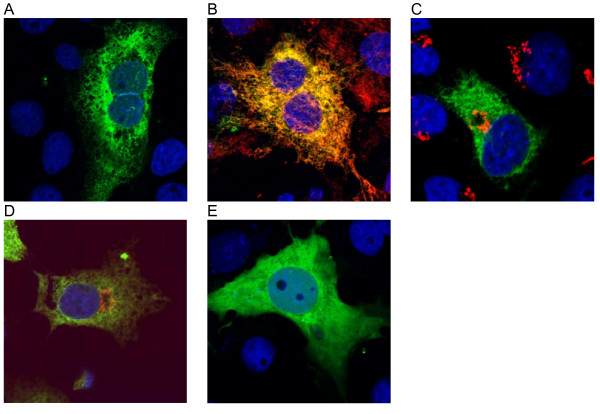
**Subcellular localization of fibin in COS-7 cells**. Confocal fluorescence images (LSM 510, Zeiss) show (A) fibin-GFP, (B) merge (yellow) of fibin-GFP (green) with the ER marker calnexin (red), (C) merge (yellow) of fibin-GFP (green) with the Golgi marker GM130 (red), (D) merge (yellow) of fibin, detected with anti-fibin-antibody-1/anti-rabbit-IgG-FITC (green) and the Golgi marker GM130 (red), and (E) Δ1-27-fibin-GFP.

To study the subcellular distribution of fibin a C-terminally GFP-tagged version was heterologously expressed in COS-7 cells. As demonstrated in Figure 6A fibin-GFP transfected cells displayed a reticular fluorescence pattern suggesting association of fibin with the ER. Co-immunostaining with an antibody against the ER-specific protein calnexin showed co-localization (Figure 6B). Only a very low signal overlap was seen with Golgi-specific protein GM130 (Figure 6C). An identical subcellular distribution was found for wild-type fibin using the mouse fibin-specific antibody (Figure 6D). Because no structural signatures of transmembrane domains were predicted we speculate that the N terminus contains a signal sequence guiding the protein to the ER. To address this hypothesis we generated an N-terminally truncated fibin-GFP construct starting with the methionine at position 28. As shown in Figure 6E this construct was homogenously distributed in the cell indicating that the deleted N terminus contained a signal sequence for ER localization.

To finally verify whether the putative ER-targeting signal is cleaved from the precursor protein we purified a fibin construct C-terminal tagged with His_6 _from COS-7 cells under denaturating conditions and sequenced the N terminus by the Edman method (see Methods). The native N terminus started with the amino acids Tyr-Phe-Asp, which correspond to the amino acid positions 19 to 21 in fibin. This indicates that fibin contains an N-terminal signal sequence of 18 amino acids which is removed by cleaving between Gly^18 ^and Tyr^19^.

### Posttranslational modifications and molecular forms of fibin

**Figure 7:**
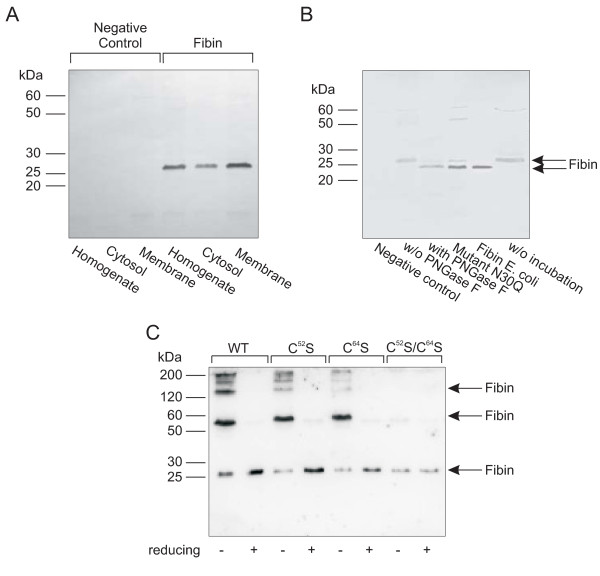
**Localization and posttranslational modification of fibin analyzed by Western blotting**. (A) To analyze the subcellular distribution of fibin cells were harvested 72 h after transfection followed by preparation of the homogenate, the cytosolic and the membrane fraction. 50 μg of protein were applied to each lane. (B) For deglycosylation experiments, fibin expressed in COS-7 cells was incubated either with or without (w/o) PNGase F or not incubated (w/o incubation). Furthermore, the fibin mutant N^30^Q lacking the N-glycosylation site, was expressed in COS-7 cells and fibin lacking the signal sequence were expressed in *E. coli *(Fibin *E. coli*). Immunoblots (A) and (B) were performed with anti-fibin-1-antibodies. (C) Wild-type (WT) fibin, and the fibin mutants C^52^S, C^64^S, C^52^S/C^64^S were expressed in COS-7 cells. After 72 h, cells were harvested and lysates were prepared. Equal amounts of protein were loaded and separated by SDS-PAGE under either non-reducing or reducing conditions. For immunoblotting the anti-fibin-2 antibody was applied. Negative controls (NC) were prepared as described under Methods.

To further substantiate the molecular architecture of fibin, protein isolation and Western blot studies were performed. Ortholog alignment and analysis of the fibin amino acid sequence by prediction software revealed an N-terminal signal sequence for ER entry and a conserved N-glycosylation site (see Additional file 2, Figure S2). The theoretical molecular weights of murine non-glycosylated full length fibin and fibin without the signal sequence were calculated as 24.8 kDa and 22.6 kDa, respectively. As demonstrated in Figure 7A, fibin appeared at 26 kDa in both the soluble and membranous fractions of transfected COS-7 cells. Furthermore, we studied fibin glycosylation, a clear marker for ER/Golgi passage. Deglycosylation with PNGase F and mutation (N^30^Q) of the potential N-glycosylation site at amino acid position 30 led to the calculated size of approximate 23 kDa (Figure 7B). Fibin heterologously expressed in *E. coli *was used as a control as it was the same size as the two deglycosylated forms (Figure 7B).

Fibin contains two conserved Cys (Cys^52^, Cys^64^) residues (see Additional file 2, Figure S2). Mutagenesis studies together with SDS-PAGE/Western blot analyses under non-reducing and reducing conditions were performed to test whether these Cys residues participate in intra- and/or intermolecular disulfide bonds. Under non-reducing conditions, specific bands were found most prominently at 26 and 56 kDa, which correspond to the monomeric and homodimeric forms of fibin, respectively (Figure 7C). The dimeric form completely disappeared under reducing conditions. None of the individual Cys mutations (Cys^52^Ser, Cys^64^Ser) promoted the monomeric form. Only the double mutant Cys^52^Ser/Cys^64^Ser resulted in a complete loss of dimer formation (Figure 7C). These findings support fibin as a dimer where two molecules are linked via two disulfide bonds between Cys^52^-Cys^52 ^and Cys^64^-Cys^64^. In the case of only one (e.g., Cys^52^-Cys^64^) or random disulfide bonds one would expect an increase of the monomeric form at least in one of the Cys mutants.

To test whether fibin also exists in non-covalent multimeric forms, size exclusion chromatography experiments were performed. Protein from fibin-myc-transfected COS-7 cells was released from microsomes by French press treatment into hypotonic buffer. The extract was applied to a Superdex 200 column and analyzed by size exclusion chromatography. Fractions were separated by SDS-PAGE under non-reducing conditions and analyzed by a Western blot. As shown in Additional file 3, Figure S3, a reasonable amount of SDS-resistant fibin multimers were found in high molecular weight fractions (>158 kDa). These bands probably represent soluble aggregates. Fibin dimers and monomers were mainly eluted in fractions below 100 kDa.

### Fibin is not secreted from COS-7 cells

**Figure 8:**
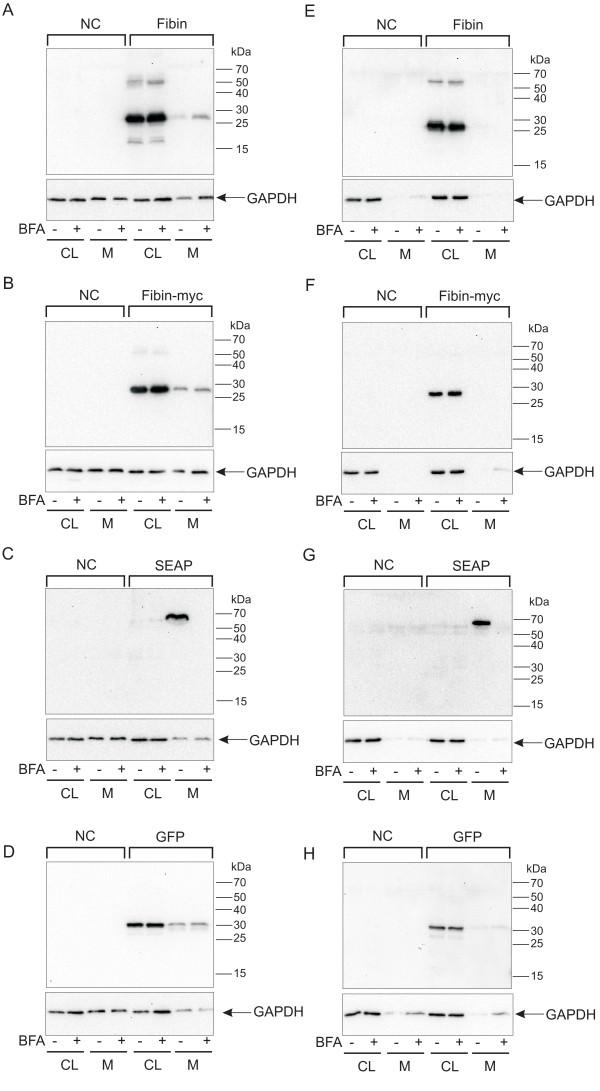
**Protein secretion studies**. COS-7 cells transfected with (A,E) fibin, (B, F) myc-tagged fibin, (C, G) SEAP, and (D, H) GFP were treated with (A, B, C, D) 250 ng/ml Brefeldin A for 24 h or with (E, F, G, H) 50 ng/ml Brefeldin A for 6 h. Serum-free medium samples were collected and concentrated by trichloroacetic acid precipitation. Cell lysates and medium were analyzed by Western blot using the anti-fibin-2 antibody, anti-myc-POD antibody, anti-AP-antibody, anti-GFP antibody and anti-GAPDH antibody as well as the respective POD-labeled secondary antibodies.

To further address the fate of fibin, serum-free medium supernatants of transfected COS-7 cells were collected, concentrated by trichloroacetic acid precipitation, and subjected to Western blot analysis. First, we analyzed the release of fibin, c-myc-tagged version of fibin, the secretory alkaline phosphatase (SEAP, positive control) and GFP (negative control) after 24 h incubation with serum-free medium. Additionally, transfected cells were treated with brefeldin A (BFA), an inhibitor of Golgi function that blocks the classical secretory pathway [8]. As shown in Figure 8 SEAP was only found in the medium but not in cell lysates. The secretion of SEAP was completely blocked by BFA (250 ng/ml). Fibin and fibin-myc were detected in the cell lysates and medium. The release of fibin and fibin-myc was not blocked by BFA. GAPDH, an endogenous cytosolic protein, and the heterologously expressed, cytosolic GFP were also found in the cell lysates and medium. These results suggested that fibin found in the medium was not actively secreted but rather passively released after cell death. To minimize unspecific release by cell damage the incubation time was reduced to 6 hours and a lower BFA concentration (50 ng/ml) was used (Figure 8E, F, G and 8H). SEAP was found in the supernatant whereas fibin was only found in the cell lysate.

## Discussion

Fibin is a structurally and evolutionary preserved vertebrate protein with no amino acid homology to known proteins (see Additional file 2, Figure S2 and Additional file 4, Table S1). Previous studies demonstrated the functional relevance of fibin during embryonic development of pectoral fin bud initiation in zebrafish and limb development in mice [1]. Indeed, fibin mRNA is embryonically expressed not only in forelimbs, but also in other tissues. Furthermore, we found reasonable fibin expression in adult tissues, with the highest expression levels in cerebellum and skeletal muscle; however, fibin mRNA was also present in most other tissues investigated (Figure 2). These data are consistent with the abundance of fibin EST in many different tissues including tumors and embryonic stages in mouse and humans indicating a role for fibin during both ontogenesis and adolescence. Recent microarray-based gene expression studies revealed an increase in fibin expression within a right cardiac ventricle during chronic pulmonary embolism [9]. This may suggest an involvement of fibin in myocardial pathogenic or remodelling processes.

The fibin gene displays a simple genomic structure without introns and a transcription start in close proximity to the translational start, AUG. Transcription regulation was studied in cell lines constitutively expressing fibin. Several compounds known to activate transcription factors which potentially bind in the promoter region of fibin (Figure 1) were tested. Lithium chloride activates the transcription factors LEF/TCF of the Wnt/β-catenin pathway via blocking GSK3β [10]. Dexamethasone, a glucocorticoid, binds to intracellular receptors which act as transcription factor. Forskolin increases cAMP via adenylyl cyclase and cAMP activates protein kinase A and the transcription factor CREB. Manganese ions can mediate effects via the NF-κB/AP-1 signalling [11,12] and the Nrf2/ARE cascade [13]. Phorbol ester activates the protein kinase C/MAP kinase pathway. In L929 cells, but not HEK293 cells, manganese ions, phorbol ester and dexamethasone induced fibin-mRNA expression with highest mRNA levels 8-16 h after stimulation (Figure 3). The genomic sequence ~836 bp upstream of the transcriptional start is sufficient to mediate basal transcription in HEK293 and L929 cells, but not in human and mouse cell lines without endogenous fibin expression (T98G, RAW264.7; Figure 4). Interestingly, and in contrast to L929 cells, HEK293 cells lack factor(s) which are required for transcription regulation upon stimulation with manganese ions, phorbol ester and dexamethasone (Figure 5).

Previous studies showed that fibin expression *in vivo *depends on the presence of wnt2b, indicating regulation of fibin expression by the Wnt/β-catenin pathway [1]. Indeed, there is a LEF-1/TCF target sequence upstream of the fibin coding sequence (Figure 1). Deletion of this sequence in our 1.52-kbp-reporter construct had no specific effect on basal luciferase activity in L929 or HEK293 cells (data not shown). No effect on luciferase activity was found when constitutively active catenin was co-expressed with the 1.5-kbp-fibin promoter construct in HEK293 and L929 cells. Using the TCF reporter gene kit (Upstate Biotechnology, Lake Placid, NY) as control the Wnt/β-catenin pathway was active in HEK293 but not in L929 cells (data not shown). Fibin expression was not increased in L929 and HEK293 cells when transfected with constitutively active β-catenin (data not shown). Moreover, lithium chloride, known to activate Wnt/β-catenin pathway, had no effect on fibin expression (Figure 3). These results indicate that the Wnt/β-catenin pathway is probably not involved in fibin regulation at least in the investigated cell line.

According to our *in silico *analysis, which predicted a signal sequence for ER entry and a glycosylation site, we experimentally demonstrated fibin localization in the ER (Figure 6) and cleavage of signal sequence and glycosylation at Asn^30 ^(Figure 7B). SDS-PAGE under non-reducing conditions and immunoblotting revealed mostly multimeric fibin formed by an intermolecular disulfide bond between the two cysteine residues, Cys^52 ^and Cys^64^, (Figure 7C). It remains unclear whether homodi- and homomultimers are the natural forms of fibin. We approached the quarternary structure of fibin by size exclusion chromatography. We found almost all the fibin in non-covalent associated multimeric complexes (see Additional file 3, Figure S3). When expressed in *E. coli*, fibin was exclusively found in inclusion bodies. Refolding with various protocols and under different reducing conditions led to a protein with high aggregation tendency (see Additional file 5, Figure S4). Expression of a thioredoxin-fibin fusion protein in *E. coli *Origami B (thioredoxin reductase/glutathione reductase deficient; Novagen Merck) resulted in lower inclusion body formation, but the soluble protein also formed multimeric complexes when analyzed by size exclusion chromatography (see Additional file 5, Figure S4). The high tendency of fibin to form multimeric complexes seems to be similar to other secretory proteins, such as adiponectin [14].

Although fibin displays properties of a secretory protein (see above), no experimental evidence for substantial fibin secretion, beyond fibin release through cell death, was found in COS-7 cells (Figure 8). This finding is in contrast to a previous study [4] which, however, lacked controls for unspecific release. Our data suggest at least three scenarios: First, fibin naturally exists as a monomer or as a homodimer formed by disulfide bridges. The insufficient secretion found in our heterologous COS-7 cell overexpression system may result from the naturally high aggregation tendency of fibin, which prevents proper trafficking to the Golgi complex. In a second scenario, fibin needs escort proteins for secretion which are not expressed in COS-7 cells. Third, fibin forms heteromers with a yet unknown protein. The lack of the partner protein would prevent proper folding and trafficking, and explains the aggregation and retention tendency of fibin in ER. There are a number of examples of the latter scenario in Wnt ligand family members [15,16], interleukin 12 (IL-12) [17,18], and Cbln3 [19]. At present, all attempts failed to prove or to reject one of these hypotheses. Yeast two-hybrid experiments using human fibin and a human kidney library revealed expected interaction partners, such as chaperones and the exocyst complex component 3, but no putative fibin subunits (data not shown). Until endogenous fibin is purified from natural sources, clarification of its functional molecular structure will remain unknown.

## Conclusions

Fibin appears to play a role not only during embryonic development but also in many adult functions. Fibin expression shows complex regulation via the signalling pathways of protein kinase C, glucocorticoids and manganese ion-activated transcriptional factors. Although fibin displays all features of a secretory protein, proper secretion presumably requires a currently unknown covalently linked or associated cofactor. Because fibin is evolutionarily old and lacks structurally related paralogs, it may have a unique functionality; conditional gene deficiency in mouse models may provide further insights into its function during different ontogenetic stages and in different tissues.

## Methods

### Generation of constructs for fibin expression in mammalian and bacterial cells

Mouse fibin cDNA was amplified from mouse genomic DNA by PCR and cloned into the mammalian expression vector pcDps. For structure-function relation studies mutations were introduced by PCR-based site-directed mutagenesis and fragment replacement strategies. For detection purposes fibin constructs were tagged C-terminal with GFP, His_6_-tag or c-myc-tag. For expression in *E. coli *fibin cDNA was cloned into the pET21c vector (Novagen Merck, Nottingham, UK) and the pET32b vector (Novagen), respectively (Detailed information see Additional file 6, Table S2).

For fibin promoter studies 5' genomic DNA fragments ranging from 0.5 to 2.5 kbp were introduced into the pGL3'basic' vector (Promega) containing the cDNA for the reporter luciferase. As positive control the SV40 promoter was cloned into the pGL3'basic' vector. A construct (Neg1520) containing 1.5 kbp (5' of the fibin coding sequence) genomic sequence in reversed orientation served as negative control.

All PCR-derived constructs were confirmed by restriction enzyme analysis and DNA sequencing.

### Real-time PCR for quantification of fibin

Total RNA was isolated from tissues, HEK293 and L929 cells using Trizol (Invitrogen, Karlsruhe, Germany). To obtain embryonic tissues at defined post coital stages mice were fertilized by superovulation according to a protocol in [20]. RNA was further purified with the SV Total RNA Isolation System (Promega, Mannheim, Germany) according to the RNA clean-up protocol including a DNAse digest. Reverse transcription of 1 μg total RNA per reaction was carried out using oligo-dT primer and Superscript^® ^II Reverse Transcriptase (Invitrogen). cDNA from 50 ng total RNA was subjected to qPCR using Platinum-SYBR Green^® ^qPCR Supermix (Invitrogen), 0.6 μM forward and reverse primers and 100 nM ROX™ (5-carboxy-X-rhodamine, passive reference dye). Oligonucleotide primers for PCR were designed with the Primer3 software http://frodo.wi.mit.edu and are indicated in Additional file 6, Table S2. All qPCR reactions were performed with the Mx3000P instrument (Stratagene, La Jolla, CA, USA) using the following protocol: 2 min at 50°C, 2 min at 95°C and 50 cycles of 15 s at 95°C, 30 s at 60°C. To confirm a single amplicon a product melting curve was recorded. The correct amplicon size and identity was confirmed by agarose gel electrophoresis and sequencing or restriction enzyme cleavage. Threshold cycle (C_t_) values were set within the exponential phase of the PCR. The house keeping gene, β_2_-microglobulin was used to normalize the C_t _values of the respective fibin transcripts (ΔC_t _= C_t _- C_t __β2-microglobulin_) [21]. Gene expression and, accordingly, regulation was statistically evaluated by subjecting the ΔC_t _values to a Student's t-test (two-tailed, unpaired, p < 0.05). Gene regulation was calculated as 2^-ΔΔCt ^and was expressed as x-fold over reference. Phorbol ester (phorbol 12-myristate 13-acetate) and forskolin additives were dissolved in DMSO.

### Cell culture and transfection

COS-7 and HEK293 cells were grown in DMEM and L929 cells were grown in RPMI medium with both containing glutamine, 10% fetal bovine serum (FBS), 100 μg/mL streptomycin, and 100 units/mL penicillin at 37°C in a humidified atmosphere of 5% CO_2 _and 95% air. For transient expression, cells were seeded into 6-well-plates (for qPCR and Western blot studies), 48-well-plates (for luciferase assays) or 10-cm dishes (for Edman sequencing) 24 h prior to transfection with Lipofectamine 2000^® ^(Invitrogen) according to the manufacturer's instructions.

### Promoter studies

For promoter studies L929 and HEK293 cells were transfected with the various 5'-fibin- luciferase constructs, washed 24 h (stimulation experiments) or 48 h (promoter length constructs) after transfection and lysed with 100 μl cell lysis buffer containing 77 mM K_2_HPO_4_, 23 mM KH_2_PO_4 _(pH 7.8), 0.2% Triton X-100, 1 mM DTT. Twenty minutes after incubation on ice 20 μl cell lysate was mixed with 100 μl luciferase buffer (20 mM Tricine pH 7.8, 2.67 mM MgSO_4_, 0.1 mM EDTA, 33.3 mM DTT, 270 μM acetyl-CoA, 530 μM adenosine triphosphate, 470 μM D-luciferin). Luciferase activity was measured in 96-well Opti-plates (PerkinElmer, Rodgau-Jügesheim, Germany) with multi-plate reader Victor2 (PerkinElmer, Rodgau-Jügesheim, Germany) as previously described [22].

Equal numbers of cells per well, identical amounts of plasmid DNA for transfection, identical volumes of lysis buffer and equal sample aliquots were used to ensure for comparable measurements. The relative luciferase activity was calculated using the following formula (MLA = Mean of luciferase activity of triplicates or duplicates):

### Generation of antibodies

To determine the cellular fate and subcellular distribution of fibin we performed studies with epitope and GFP-tagged fibin constructs. We also generated anti-fibin antibodies suitable to detect non-modified fibin. Thus, anti-fibin antiserum was obtained by immunizing rabbits with the peptide RLGRLKSDYLEGGAQ corresponding to amino acid position 200 to 214 of mouse fibin. The peptide was conjugated with keyhole limpet hemocyanine (prepared by Dr. Sven Rothemund, IZKF Leipzig). To obtain polyvalent antiserum mouse fibin was expressed in *E. coli*. The isolated inclusion bodies were purified by size exclusion chromatography followed by preparative SDS-PAGE. After dialysis against 20 mM Hepes pH 7.5 for 24 h, fibin was incubated with 0.002 × volume 50% glutardialdehyde (Sigma, St. Louis, USA) for 5 min at 25°C. The reaction was stopped by addition of 0.1 volume of 1 M Tris-HCl pH 7.4, followed by dialysis against 20 mM Hepes pH 7.4 for 24 h. Efficiency of cross linking was revised visually by SDS-PAGE. For immunization either 200-300 μg keyhole limpet hemocyanine-coupled peptide or approximately 700 μg cross-linked fibin were mixed with an equal volume of complete Freund's adjuvant to immunize rabbits. After eight weeks rabbits were boostered and blood samples were collected. The specific antibodies were purified by affinity chromatography using CNBr-activated sepharose 4B (GE Healthcare Biosciences, Uppsala, Sweden) coupled with the peptide or mouse fibin applying manufacturer's protocol. Antibodies generated by immunization with peptide conjugate and the whole protein were referred to as anti-fibin-1-antibodies and anti-fibin-2-antibodies, respectively. Both antibodies were highly specific in Western blot analyses using cell extracts from transiently transfected COS-7 cells and transformed *E. coli*.

### Immunofluorescence studies und subcellular distribution

For immunofluorescence studies COS-7 cells were seeded into 6-well plates containing cover slips. 72 h after transfection cells were washed, fixed with 4% paraformaldehyde in PBS and permeabilized with 0.5% Triton X-100 in PBS. Cells were incubated with the antibodies indicated (Figure 5) and the following dilutions were used: rabbit anti-fibin antisera (1:500 dilution), rabbit anti-calnexin antibody (SIGMA, dilution 1:200) and mouse anti-GM130 antibody (BD Biosciences, developed in mouse, dilution 1:250). After washing, cells were incubated with anti-mouse IgG-TRITC conjugate (SIGMA, dilution 1:2,000) or anti-rabbit IgG-FITC conjugate 1:4,000 (SIGMA, St. Louis, USA) diluted. After washing again, cells were incubated with 4', 6-diamidino-2-phenylindoldihydrochloride (DAPI, Merck) for 2 min and washed. Finally, cover slips were mounted with Fluoromount (Molecular Probes, Eugene, USA). For preparation of cytosolic and membrane fractions COS-7 cells transfected for 48 h with fibin-pcDps construct were homogenized in ice-cold buffer containing 50 mM Tris-HCl pH 7.4, 250 mM sucrose, 1 mM EDTA and protease inhibitor mix (Sigma) by ultra sonication. The homogenates were centrifuged at 1,000 *g *and 4°C for 5 min to remove cell debris. The supernatant was centrifuged at 100,000 *g *and 4°C for 1 h. The resulting supernatant was collected and the pellet was resuspended with homogenization buffer. Protein concentrations of the fractions were estimated by the Bradford method.

### Edman sequencing and PNGase F treatment of fibin

COS-7 cells were transfected with fibin-His_6_-pcDps and harvested 72 h after transfection. Cells were lysed (50 mM Tris-HCl pH 7.4, 8 M urea, 150 mM NaCl), ultra sonication treated and centrifuged at 20,000 *g *for 20 min at 10°C. The supernatant was incubated with 1 ml of Ni^2+^-IDA-Sepharose in a closed column for 30 min at room temperature under slow spinning. Then, the column was washed (50 mM Tris-HCl pH 7.4, 8 M urea, 150 mM NaCl, 20 mM imidazole), the protein was eluted (50 mM Tris-HCl pH 7.4, 8 M urea, 150 mM NaCl, 200 mM imidazole) and precipitated with trichloroacetic acid. The pellet was resuspended in sample buffer, boiled and subjected to SDS-PAGE. After electroblotting to PVDF membrane (PALL corporation, Ann Arbor, USA) using 100 mM CAPS buffer pH 8.0 with 20% methanol and Coomassie blue staining the protein bands from three lanes were cut and used for N-terminal sequencing according to the Edman procedure using the Protein Sequencer 473A (Applied Biosystems, Foster City, CA).

For protein deglycosylation samples were treated with PNGase (Sigma, St. Louis, USA) according to the manufacturer's protocol. After incubation time for 2 h at 37°C reaction was stopped at 94°C for 5 min.

### Western blot analysis

SDS-PAGE was performed under reducing conditions, if not otherwise indicated, with 12.5% (w/v) polyacrylamide gels using the Mini-Protean 3 (Bio-Rad, Munich, Germany). After electrophoretic separation proteins were electroblotted onto Hybond-ECL nitrocellulose membrane (Amersham GE Health Sciences). The membranes were blocked for 30 min at room temperature. Then, blots were incubated with primary antibodies for 2 h at room temperature or overnight at 4°C. Anti-c-myc-POD antibody (Miltenyi Biotec, Bergisch-Gladbach, Germany), anti-alkaline phosphatase mouse antibody A-10 (Santa Cruz Biotechnology, USA) with a dilution of 1:200, anti-GAPDH rabbit antibody (Sigma, St. Louis, USA) 1:2,000 diluted, and 1 μg/ml affinity purified anti-fibin-1 or anti-fibin-2 antibodies (rabbit) were used. After washing with PBS/Tween (0.05%) anti-rabbit-IgG conjugated with horseradish-peroxidase (1:3,000 dilution, Dianova, Hamburg, Germany) or anti-mouse-IgG conjugated with POD (Sigma) diluted 1:5,000 were used as secondary antibodies. After 1 h of incubation and washing blots were developed with diaminobenzidine tetrahydrochloride or with a chemoluminescence kit (Thermo Scientific, Rockford, USA).

For negative controls COS-7 cells were transfected with GFP-pcDps (Figure 7) or with the empty pcDps vector (Figure 8).

## Abbreviations

DMEM: Dulbecco's modified Eagle medium; DMSO: dimethyl sulfoxide; ER: endoplasmic reticulum; EST: expressed sequence tag; FBS: fetal bovine serum; qPCR: quantitative real-time PCR analysis; WT: wild type;

## Authors' contributions

JL and TH performed bioinformatics and expression analyses. Promoter and expression regulation studies were performed by CS. TH and TS wrote the manuscript. All authors read and approved the final manuscript.

## Supplementary Material

Additional file 1 **Figure S1 Putative transcript lengths and transcription starts**. Click here for file

Additional file 2 **Figure S2 Conservation of amino acid positions, hydrophobicity, and alpha helix prediction for fibin**. Click here for file

Additional file 3 **Figure S3 Size-exclusion chromatography of cellular fibin-c-myc**. Click here for file

Additional file 4 **Table S1 Fibin orthologs investigated in this study**. Click here for file

Additional file 5 **Figure S4 Expression of fibin in *Escherichia coli***. Click here for file

Additional file 6 **Table S2 Primers used in this study**. Click here for file
