# Supplementary material for: Characterization of the expression, promoter activity and molecular architecture of fibin
Source: BMC Biochem. 2011 May 26;12:26. doi: 10.1186/1471-2091-12-26 (PMC3115872; doi:10.1186/1471-2091-12-26)
Supplement: Additional file 1 — Figure S1 Putative transcript lengths and transcription starts. [file 1471-2091-12-26-S1.PDF]

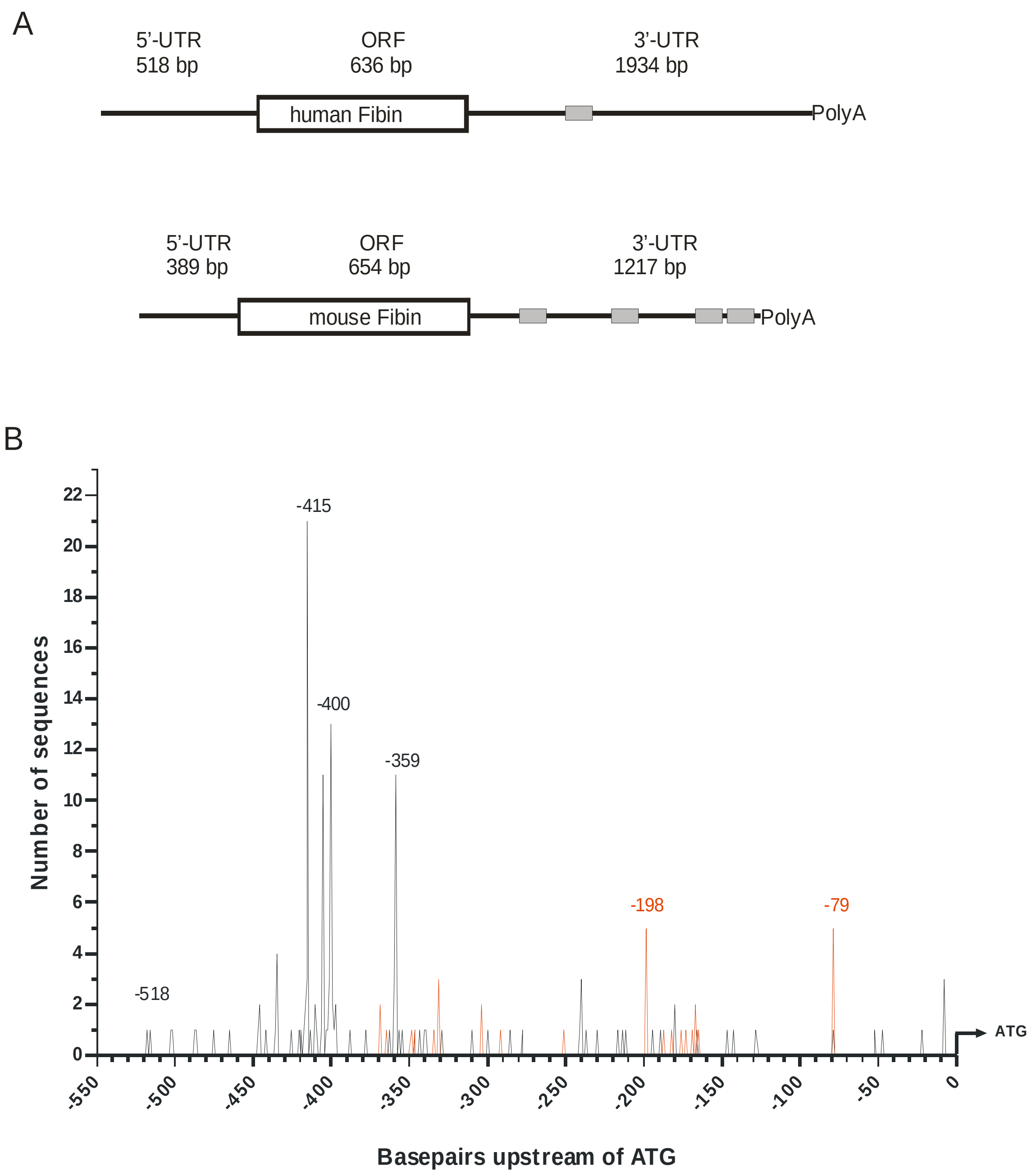

Figure S1 Putative transcript lengths and transcription starts.

(A) Mouse and human ESTs were compared with the respective genomic sequences to determine structural elements and the length of fibin transcripts. The human and mouse transcripts have a length of 3089 and 2260 nucleotides, respectively. Polyadenylation sequence signals (ATTAAA and AATAAA) are shown as grey boxes. (B) The number of sequences and the 5' position projected to the genomic sequence were determined from 140 human ESTs (black) and 27 mouse ESTs (red) to estimate the translational starting point.
