# Supplementary material for: Characterization of the expression, promoter activity and molecular architecture of fibin
Source: BMC Biochem. 2011 May 26;12:26. doi: 10.1186/1471-2091-12-26 (PMC3115872; doi:10.1186/1471-2091-12-26)
Supplement: Additional file 2 — Figure S2 Conservation of amino acid positions, hydrophobicity, and alpha helix prediction for fibin. [file 1471-2091-12-26-S2.PDF]

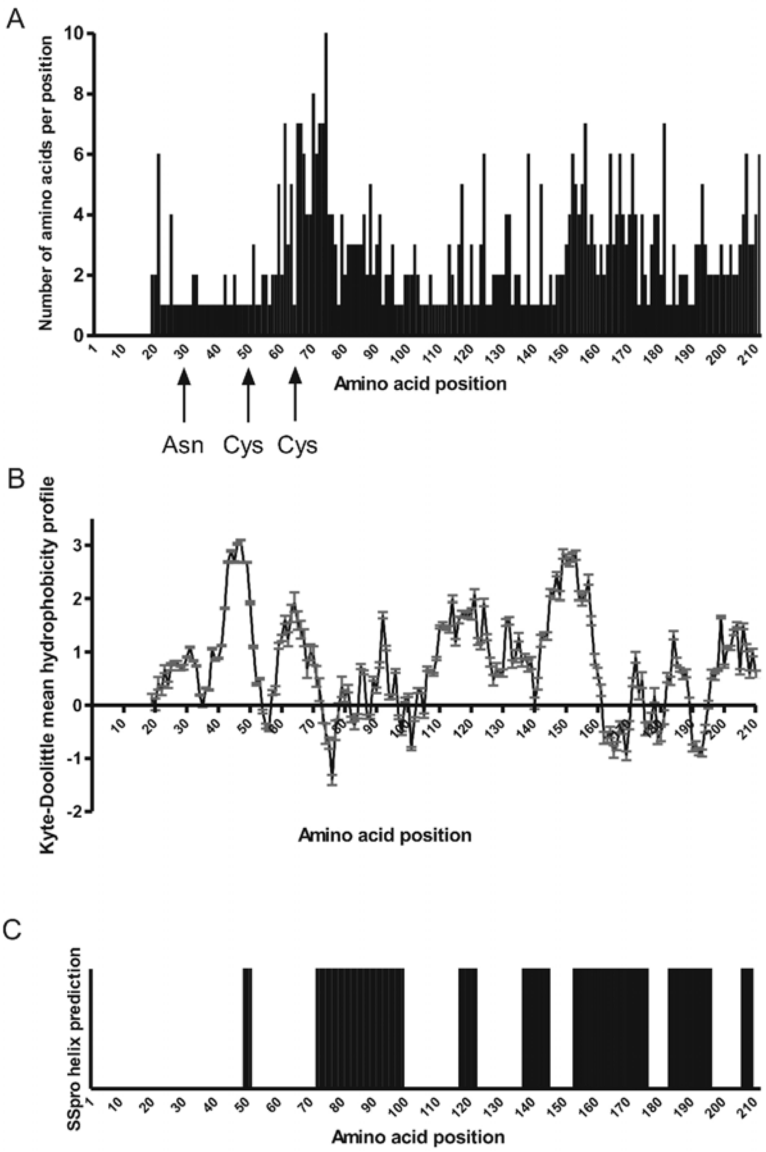

Figure S2 Conservation of amino acid positions, hydrophobicity, and alpha helix prediction for fibin.

(A) Structural conservation: 40 ortholog sequences were aligned with the ClustalW program. The number of different amino acids per position was determined. Indicated asparagine is conserved in all species and predicted as putative glycosylation site. Indicated cysteines are also conserved in all species. (B) Hydrophobicity analysis: 15 representative orthologs (fugu, zebrafish, stickleback, western clawed frog, red-throat anole, chicken, zebra finch, platypus, west european hedgehog, little brown bat, nine banded armadillo, mouse, elephant, dog, human) were analysed by Kyte-Dolittle hydrophobicity algorhythm. Data are shown as means

± SEM. (C) Helix prediction: Based on the human amino acid sequence regions with a potential alpha helical structure were predicted by SSpro.
