# Supplementary material for: Characterization of the expression, promoter activity and molecular architecture of fibin
Source: BMC Biochem. 2011 May 26;12:26. doi: 10.1186/1471-2091-12-26 (PMC3115872; doi:10.1186/1471-2091-12-26)
Supplement: Additional file 3 — Figure S3 Size-exclusion chromatography of cellular fibin-c-myc. [file 1471-2091-12-26-S3.PDF]

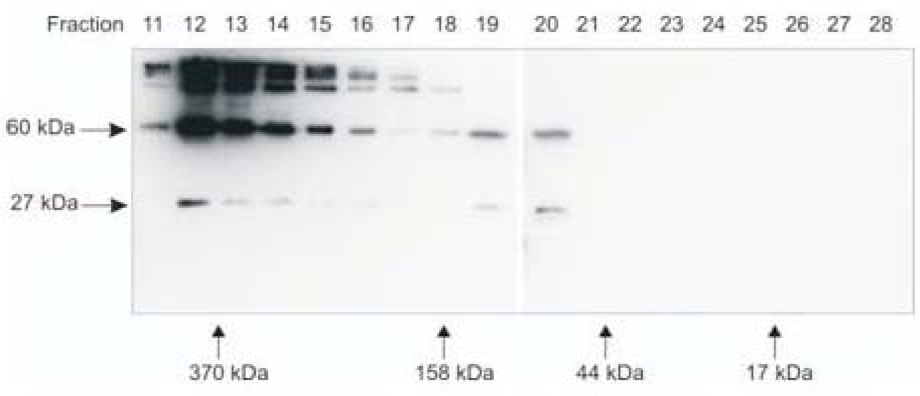

Figure S3 Size-exclusion chromatography of cellular fibin-c-myc.

Fibin-c-myc transfected cells were resuspended in 50 mM Tris-HCl pH 7.4, 1 mM EDTA and protease inhibitor mix (Sigma), followed by cell disruption with French press and ultra sonication. The homogenate was centrifuged at 100,000 *g* for 1 h at 4 °C. The resulting supernatant (2 ml) was applied to a Superdex 200 column 16/60 (Amersham GE Healthcare) and eluted with 160 ml 50 mM Tris-HCl pH 7.4, 200 mM NaCl at a flow rate of 1 ml/min in 4-ml fractions. For Western blot analysis, equal aliquotes of fractions were concentrated by trichloroacetic acid precipitation
