# Supplementary material for: Characterization of the expression, promoter activity and molecular architecture of fibin
Source: BMC Biochem. 2011 May 26;12:26. doi: 10.1186/1471-2091-12-26 (PMC3115872; doi:10.1186/1471-2091-12-26)
Supplement: Additional file 4 — Table S1 Fibin orthologs investigated in this study. [file 1471-2091-12-26-S4.PDF]

Table S1 Fibin orthologs investigated in this study. Protein sequences were obtained through translation of open reading frames from NCBI database blastN* search or from alignment of nucleotide sequences received from trace archive database. Identities were determined through amino acid sequence alignment (MegAlign, Lasergene 7).

Classes and species	Length (Amino acids)	Identity to human (%)
**Chondrichthyes**		
Callorhinchus milii	N-terminal sequence data incompletely	--
**Osteichthyes**		
Takifugu rubripes	205	63.9
Tetraodon nigroviridis	205	62.9
Gasterosteus aculeatus	207	62.1
*Danio rerio** NM_001017873	210	62.9
**Amphibia**		
*Xenopus tropicalis** XM_002938894	203	67.6
**Reptilia**		
Anolis carolinensis	230	67.9
**Aves**		
*Gallus gallus** XM_424544	206	71.4
*Taeniopygia guttata** XM_002195064	207	69.4
**Mammalia**		
*Ornithorhynchus anatinus** XM_001511157	212	82.1
*Monodelphis domestica** XM_001380133	211	88.7
Macropus eugenii	211	88.2
Erinaceus europaeus	212	92.8
Myotis lucifugus	211	93.4
Pteropus vampyrus	211	95.8
Dasypus novemcinctus	211	94.8
Dipodomys ordii	211	92.9
Spermophilus tridecemlineatus	211	92.0
Cavia porcellus	211	86.3
*Mus musculus** AB236893	217	92.9
*Oryctolagus cuniculus** XM_002709021	211	95.8
Sorex araneus	211	93.9
*Rattus norvegicus** NM_001025042	217	92.0
Tupaia belangeri	211	95.3
Ochotona princeps	211	96.2
Loxodonta africana	211	96.2

Bos taurus NM_001015541*	211	96.2
Equua caballus NM_001099443*	211	96.7
Sus scrofa	211	96.7
Tursiops truncatus	211	94.8
Felix catus	211	96.7
Canis familiaris	211	95.8
Otolemur garnettii	212	97.2
Tarsius syrichta	212	93.4
Macaca mulatta XM_001090650*	211	98.6
Papio hamadryas	211	98.6
Callithrix jacchus XM_002755124*	211	97.2
Pongo pygmaeus abelii	211	98.6
Pan troglodytes XM_508336*	211	100.0
Homo sapiens NM_203371*	211	100.0

Additional information

To evaluate the evolutionary trait and origin of fibin, public data base were mined and fibin sequences from 40 species were retrieved from various vertebrate species including mammals, marsupials, monotremes, birds, reptiles, amphibians, teleost fishes and chondrichthyes (see above). This indicates that fibin exists in the vertebrate genome since more than 450 million years. In ray-finned fishes, e.g., *Danio rerio* and *Tetraodon nigroviridis*, we identified a fibin paralog which probably arose by whole genome duplication. Fibin paralogs were not found in tetrapods. Fibin-like sequences were not found in lamprey (primitive chordate), sea urchin and the known genomes of *Caenorhabditis elegans*, *Drosophila melanogaster*, and *Anopheles gambiae*.

Structural comparison of the retrieved orthologs revealed that fibin is highly conserved during vertebrate evolution (62 % amino acid identity between fish and mammals). The amino acid length varies between 203 (clawed frog) and 230 (anole). Most of the mammalian fibin orthologs comprised 211 amino acids. No significant homology to any other proteins or protein domains was found with common bioinformatic tools. Alignment of orthologs revealed several conserved regions, e.g. an amino acids block (position 20-58) in close proximity to the N terminus. Secondary structure and posttranslational modification prediction tools indicated no transmembrane domains, coiled coil regions, O-glycosylation, prenylation, amidation, myristoylation and GPI anchoring sites but predicted a conserved N-terminal signal sequence of 18 amino acids and one N-glycosylation site (Asn^{30}).
