# Supplementary material for: Characterization of the expression, promoter activity and molecular architecture of fibin
Source: BMC Biochem. 2011 May 26;12:26. doi: 10.1186/1471-2091-12-26 (PMC3115872; doi:10.1186/1471-2091-12-26)
Supplement: Additional file 5 — Figure S4 Expression of fibin in Escherichia coli. [file 1471-2091-12-26-S5.PDF]

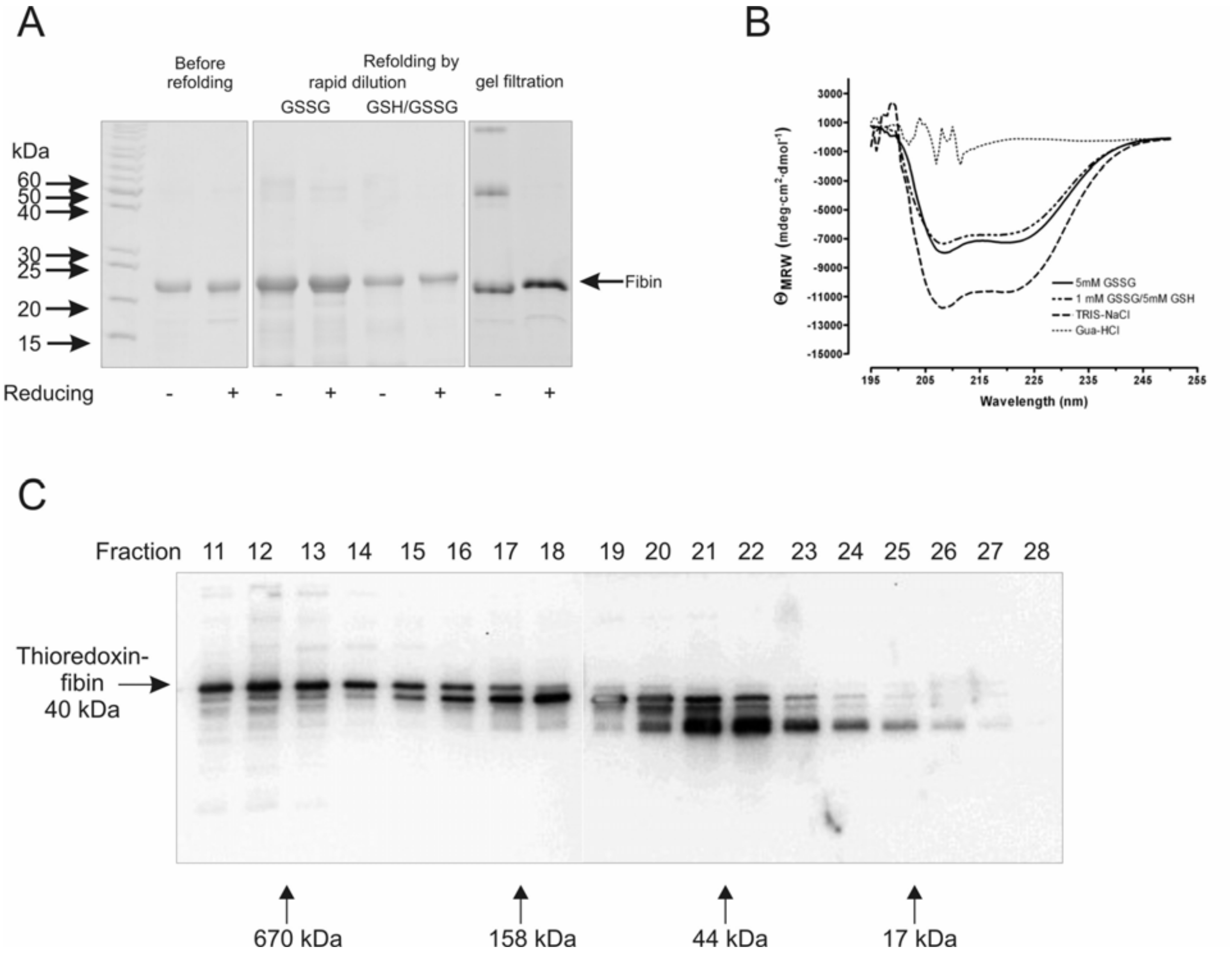

Figure S4 Expression of fibin in *Escherichia coli*.

The strain BL21 DE3 and the strain Origami B were transformed with the pET21c-fibin construct and the pET32b-thioredoxin-fibin construct respectively. (A) Fibin inclusion bodies expressed in *E. coli* BL21 were refolded in 50 mM Tris-HCl, 600 mM arginine hydrochloride, 1 mM EDTA pH 8.2 either in the presence of 5 mM oxidized glutathione or 5 mM reduced glutathione/1 mM oxidized glutathione by the rapid dilution method for 24 h at 4°C. In another protocol refolding was performed by column gel filtration in 50 mM Tris-HCl pH 7.4, 200 mM NaCl. Samples were analysed by SDS-PAGE under reducing and non-reducing conditions and stained with Coomassie blue. (B) Refolded fibin was analysed by circular dichroism spectroscopy using the J-715 spectrometer (Jasco Corp.) (20° C, wavelength 190–250 nm) (C) Soluble fraction of thioredoxin-fibin was prepared and applied to a Superdex 200

column. Fractions were collected and analysed by SDS-PAGE under non-reducing conditions and Western blot. Arrows indicate the peaks of marker proteins used for column calibration. Additional methods see below.

Expression and refolding of fibin

The *Escherichia. coli* strain BL21 DE3 and the *E. coli* strain Origami B transformed with the pET21c-fibin construct and the pET32b-fibin construct respectively were grown overnight in LB medium with ampicillin at 37 °C. This cultures were used to inoculate 800 ml batches which were grown until an $OD_{600\ nm}$ of 0.6. Protein expression was induced by addition of 0.8 mM isopropyl-ß-thiogalactoside. Incubations were continued for 4 h at 37 °C (*E. coli* BL21) or at 15 °C (*E. coli* Origami B). Then, cells were centrifuged and the cell pellet was resuspended in 30 ml of 100 mM Tris-HCl pH 7.0, 1 mM EDTA. After incubation with 5 mg lysozyme for 30 min at 4° C cells were disrupted using a French press (SLM instruments Inc. Aminco). The *E. coli* Origami B homogenate was centrifuged for 10 min at 30,000 *g* and 4° C and the soluble fraction was used for size exclusion chromatography. In order to isolate inclusion bodies the *E. coli* BL21 suspension was treated with DNase (10 µg/ml final concentration) and $MgCl_2$ (3 mM final concentration) for 30 min at room temperature. After addition of 0.5 volumes of 60 mM EDTA, 6 % Triton X-100, 1.5 M NaCl pH 7.0 the suspension was stirred for 30 min at 4° C followed by centrifugation at 30,000 *g* for 10 min at 4 C. The pelleted inclusion bodies were resuspended with 100 mM Tris-HCl pH 7.0, 20 mM EDTA and centrifuged (30,000 *g*, 4 °C, 10 min). These washing steps were repeated three times. Inclusion bodies were solubilized with 100 mM Tris-HCl pH 8.0, 1 mM EDTA, 100 mM dithiothreitol, 6 M guanidine HCl (80 mg inclusion bodies/ml) and incubated for 2 h at room temperature. After centrifugation inclusion bodies were applied to a Sephadex G-25 column and eluted with 50 mM MES pH 5.0, 1 mM EDTA, 6 M guanidine HCl. Protein concentration used for refolding experiments were approximately 20 mg/ml.

Solubilized inclusion bodies were added by rapid dilution to 6 ml of refolding buffer (final protein concentration 200 µg/ml) containing 50 mM Tris-HCl, 600 mM arginine hydrochloride, 1 mM EDTA pH 8.2 either 5 mM oxidized glutathione or 1 mM oxidized glutathione/5 mM reduced glutathione. After 24 hours at 4 °C samples were separated by size exclusion chromatography using a column HiLoad 16/60 Superdex 200 (GE Healthcare, Freiburg, Germany) with 50 mM Tris-HCl pH 7.4, 200 mM NaCl. Two peaks were found at 600 kDa and 90 kDa. The fractions of the second peak were pooled, dialysed against 50 mM Tris-HCl pH 7.4 for 24 h and concentrated by ultrafiltration (50 ml-Amicon chamber) to 200

to 400 μg/ml. Refolded protein was analysed by circular dichroism (CD) spectroscopy using the J-715 spectrometer (Jasco Corp.) (20° C, wavelength 190– 250 nm). In another protocol solubilized inclusion bodies were directly applied to the Superdex 200 column and eluted with 50 mM Tris-HCl pH 7.4, 200 mM NaCl at a flow rate of 1 ml/min. The peak fraction at 90 kDa was dialysed against 50 mM Tris-HCl pH 7.4 and analysed in CD spectroscopy.
