# Supplementary material for: Characterization of the expression, promoter activity and molecular architecture of fibin
Source: BMC Biochem. 2011 May 26;12:26. doi: 10.1186/1471-2091-12-26 (PMC3115872; doi:10.1186/1471-2091-12-26)
Supplement: Additional file 6 — Table S2 Primers used in this study. [file 1471-2091-12-26-S6.PDF]

Table S2 Primer used in this study.

The cloning of the several constructs is shown below.

Primer	Sequence (5’-3’)
BC-s (qPCR)	GGTTTCTTTTGCCACCTGTGT
BC-1-as (qPCR)	TCCATCAGGCACGAAGTAATG
ß-MG-1 s (qPCR)	GCTATCCAGAAAACCCCTCAAA
ß-MG-1 as (qPCR)	GGCGGGTGGAACTGTGTTA
mBC-3-s (qPCR)	GGGCACAGAAGACAGGCTAAT
mBC-3-as (qPCR)	TCGAAAGATGACTTCAGACGTG
ß-MG-ex-s (qPCR)	CCTCACATTGAAATCCAAATGC
ß-MG-ex-as (qPCR)	AGAAAGACCAGTCCTTGCTGAA
hu-qBC1-s (qPCR)	AAATCCCTCACTGAGCTGGAG
hu-qBC1-as (qPCR)	GACCAGCATTCCCAGAAAGTC
hu-beta-MG-s (qPCR)	AGTATGCCTGCCGTGTGAAC
hu-beta-MG-as (qPCR)	GCAAGCAAGCAGAATTTGGA
huBC-3'-s (qPCR)	TGGCCCTCACCATTAGGAG
huBC-3'-as (qPCR)	GGCTGATTTGATTCCCTCTTTC
hu-ex-beta-MG-s (qPCR)	TTCATCCATCCGACATTGAAG
hu-ex-beta-MG-as (qPCR)	CCAGTCCTTGCTGAAAGACAA
mNM026FL-2-s	TGGACTAAAACGCTTCTC
mNM026FL-2-as	AATATTAGCCTGTCTTCTGTG
HA-mNM0262 EcoRI-1-s	GCGAATTCCCCACCATGTACCCCTACGACGTCCCCGACTACGCCGT GTTCCCGAAGTTGATCTGG
mNM0262- Stopp SpeI-1-as	GGACTAGTTAGCCTGTCTTCTGTGC
mNM0262 GFP EcoRI-1-s	GCGAATTCCCCACCATGGTGTTCCCGAAGTTGATC
mNM0262 GFP EcoRV-2-as	CGATATCGCCTGTCTTCTGTGCCCCACCCTC
pcDps-s	GTGCAAATCAAAGAACTGCTCCTC
BC-N30Q-s	CCCAGAAATGTCTCAGGGGACTTTGCATC
BC-N30Q-as	GATGCAAAGTCCCCTGAGACATTTCTGGG
026-His-stop-SpeI-as	GGACTAGTCAATGATGATGATGATGATGGTCGCCTGTCTTC TGTGCCCCACCCTC
GFP-pcDps-1-as	ACTTGTGGCCGTTTACGTCGC
BC-myc-stop-SpeI-as	GGACTAGTCACAGATCTTCTTCAGAAATAAGTTTTTGTTCGC CTGTCTTCTGTGCCCCACCC
delmNM026_ 28-217 EcoRI-s	CAGCGAATTCCCCACCATGTCTAATGGGACTTTGCATCA
YFD-BC-pET21-1-s	CTGCATATGTACTTTGATGGCCCCCTGTACCCAG
YFD-BC-22-XhoI-as	TGCTCGAGGCCTGTCTTCTGTGCCCCAC
EcoRV-YFD-BC-s	CTGATATCTACTTTGATGGCCCCCTGTACCCAG
BamHI-32-BC-1-s	CGCGGATCCGTACTTTGATGGCCCCCTGTA
mNM026 pET EcoRI-1-as	CTCTCGAATTCTTAGCCTGTCTTCTGTG
mBC-Cys1-as	AGAGCAGCTGGCTTTTCTCG
mBC-Cys1-s	CGAGAAAAGCCAGCTGCTCT
mBC-Cys2N-as	AGCTGCGCCTGCGATCACTCA
mBC-Cys2N-s	TGAGTGATCGCAGGCGCAGCT
Primer_520bp_s_Nhe1	ATCGCTAGCAGGTTCTTTTCCAAACTTGTTCCCTG
P590bp_s_Nhe1	ATCGCTAGCACCAGGGTTTTTGTGGTGGA
P1500-2 NheI-s	TATGCTAGCATTACAGACGTGAGCCACCAC
P1500-3 neg BglII-s	GATAGATCTATTACAGACGTGAGCCACCAC
P0 neg NheI-as	CGTGCTAGCTCTGGATATTCAGGATGGCC
P2500-2 NheI-s	TCGCTAGCGCAAGTGGGGAGCAAGTAGT
P o BglII-as	AGCAGATCTTCTGGATATTCAGGATGGCC

Cloning of the fibin expression constructs

Fibin was amplified from genomic mouse DNA by PCR using gene specific primers (mNM026FL-2s/mNM026FL-2as). The PCR product was cloned into the pCR®2.1-TOPO® vector (Invitrogen, Karlsruhe, Germany). Sequencing of the open reading frame confirmed the murine fibin mRNA sequence in the data base (accession numbers AK159648, AB236893 and BC27250). For monitoring cellular expression fibin was subcloned into the mammalian expression vector pcDps (via *EcoR*I/*Blp*I) and tagged with an N terminal HA-tag (HA-fibin-pcDps), inserted downstream the signal peptide and with a C-terminal GFP (fibin-GFP-pcDps). The generation of these constructs was performed by PCR and fragment replacement using the primer pairs HA-mNM0262 EcoRI-1s/mNM0262-Stopp SpeI-1as (HA-fibin-pcDps) and mNM0262 GFP EcoRI-1s/mNM0262 GFP EcoRV-2as (fibin-GFP-pcDps). For purification purposes fibin was tagged with a C-terminal His_6-tag (primer 026-His-stop-SpeI-as) and a c-myc-tag (primer BC-myc-stop-SpeI-as). The N^{30}Qfibin-GFP-pcDps construct was generated by an overlap PCR/fragment replacement strategy using the mutagenesis primer pairs BC-N^{30}Q-s/GFP-pcDps-1as and pcDps-1s/BC-N^{30}Q-as and the restriction sites *EcoR*I/*Mlu*I. The Cys^{52}Ser-fibin, Cys^{64}Ser-fibin and $Cys^{52/64}$Ser-fibin mutants were generated by an overlap PCR/fragment replacement strategy using the primer pairs mBC-Cys1-s/mBC-Cys1-as and mBC-Cys2N-s/mBC-Cys2N-as.

For *E. coli* expression and purification via a C-terminal His_6-tag, fibin coding sequence was amplified with the primer pair YFD-BC-pET21-1s/YFD-BC-22-XhoI-as and inserted into the pET21c vector (Novagen, Merck, Nottingham, UK) using the restriction sites *Nde*I or *Xho*I. To generate a fibin fusion protein exportable into the periplasmic space fibin cDNA was amplified with the primer pair EcoRV-YFP-BC-s/mNM026 pET EcoRI-1as and cloned into the pET39b vector (Novagen) using the restriction sites *Sca*I and *EcoR*I. To express fibin without any tags the fibin-His_6-pET21c construct and the Dsba-fibin-pET39b construct were digested with *Nco*I/*Xho*I. The product excised from Dsba-fibin-pET39b was cloned into the digested fibin-His_6-pET21c vector. The thioredoxin-fibin construct was generated with the primer pair BamHI-32-BC-1-s/mNM026 pET EcoRI-1as and cloned into the pET32b vector (Novagen) after *BamHI/EcoRI* digest. The correctness of all clones was verified by sequencing.
